# Design of Double-Layer Electrically Extremely Small-Size Displacement Sensor

**DOI:** 10.3390/s21144923

**Published:** 2021-07-20

**Authors:** Yi-Dong Wang, Feng-Yuan Han, Jin Zhao, Zi-Wen Zhang, Di Wang, Yun-Hua Tan, Pu-Kun Liu

**Affiliations:** Department of Electronics, Peking University, Beijing 100871, China; wangyidong@pku.edu.cn (Y.-D.W.); typhooneyes@pku.edu.cn (F.-Y.H.); zhaojin2019@pku.edu.cn (J.Z.); zhangziwen@pku.edu.cn (Z.-W.Z.); w_dd@pku.edu.cn (D.W.); pkliu@pku.edu.cn (P.-K.L.)

**Keywords:** microwave sensors, metamaterials, electrical small size, displacement measurement, mutual coupling

## Abstract

In this paper, a displacement sensor with an electrically extremely small size and high sensitivity is proposed based on an elaborately designed metamaterial element, i.e., coupled split-ring resonators (SRRs). The sensor consists of a feeding structure with a rectangular opening loop and a sensing structure with double-layer coupled SRRs. The movable double-layer structures can be used to measure the relative displacement. The size of microwave displacement sensors can be significantly reduced due to the compact feeding and sensing structures. By adjusting the position of the split gap within the resonator, the detection directions of the displacement sensing can be further expanded accordingly (along with the *x*- or *y*-axis) without increasing its physical size. Compared with previous works, the extremely compact size of 0.05*λ*_0_ × 0.05*λ*_0_ (*λ*_0_ denotes the free-space wavelength), a high sensitivity, and a high quality factor (*Q*-factor) can be achieved by the proposed sensor. From the perspective of the advantages above, the proposed sensor holds promise for being applied in many high-precision industrial measurement scenarios.

## 1. Introduction

In recent years, the use of metamaterials in sensors has attracted great attention from the scientific community. Metamaterials can interact dramatically with the surrounding dielectric environment due to the enhanced properties of local electromagnetic fields, thereby providing a new solution for low sensitivity and low resolution of traditional sensors. Moreover, the subwavelength-size characteristic of metamaterial elements is conducive to the integration and miniaturization of sensors. Owing to these advantages, metamaterials-based sensors have been developed rapidly, and their applications are becoming more and more diversified, including biological component sensor [1], gas concentration sensor [2], liquid content sensor [3], angular displacement sensor [4,5,6], displacement sensor [7,8,9,10,11,12,13,14,15], and permittivity sensor [16].

Displacement sensors are widely applied to bridge crack detection, liquid level detection, elevator position control system, automatic door control system, landslide detection, and other industrial automation fields. Considering its applications, many microwave sensors for measuring displacement have been proposed with regard to its advantages of the low cost of microwave devices, the easy measurement of the generated signals, the wide range of applications, and the compact structures [7,8,9,10,11,12,13,14,15]. Recently, displacement sensors based on the signals of resonant depth have been proposed, such as open-ended transmission lines loaded with split-ring resonators (SRRs) in [10] and electromagnetic bandgap (EBG) consisting of periodic objects in [11,12]. The sensing signals of other displacement sensors are obtained by evaluating the shift of resonance peaks. One example is a two-dimensional displacement sensor based on a microstrip line loaded with movable broadside-coupled SRRs (BC-SRRs). The displacement-induced break of symmetry can lead to a shift of the resonant frequency of the sensor [8]. Moreover, in another example, a metallic patch printed on a movable substrate is utilized to modify the length of slots on a defected ground structure (DGS), thereby achieving a sensitive displacement sensor [9].

Although the above-mentioned displacement sensors possess good sensitivity, the structure is still bulky. The SRR metamaterial elements in References [8,10] are helpful to reduce the size of the sensor, whereas the guided wave structures exciting the metamaterial elements make the overall size of the sensor large. In this paper, a single-port miniaturized displacement sensor is proposed based on double-layer coupled SRRs, which are excited by an electrically small rectangular feeding loop without the need for additional matching networks. The displacement signal is measured in terms of the resonant frequency shift, which is induced by the variation of the coupling between the double-layer SRRs [17,18,19,20]. In contrast to the above-mentioned microwave displacement sensors, the advantages of the proposed one are extremely compact and high-*Q*-factor. Meanwhile, the sensor also maintains high sensitivity and high linearity. In addition, the sensing method has strong robustness and functional expansibility, which broadens the application prospect and provides a design approach for the microwave sensing field.

## 2. Design

Microwave sensors take the microwave signal as the measurement quantity, including the reflective and incident characteristics in microwave regions. To further improve the sensing abilities, the microwave sensor needs to be designed with a smaller electrical size, larger sensitivity, higher accuracy, and better resonance performance, so that the sensor can be more easily integrated and generate a more easily resolved measurable signal. Generally, microwave sensors can be divided into sensing structures and feed excitation structures. In order to realize miniaturization design, two aspects of sensing structure and feed excitation structure are considered. Among them, the sensing structure utilizes a deep sub-wavelength size metamaterial element resonator, and the feed excitation structure utilizes a magnetic dipole loop which can efficiently excite the metamaterial element.

**Figure 1:**
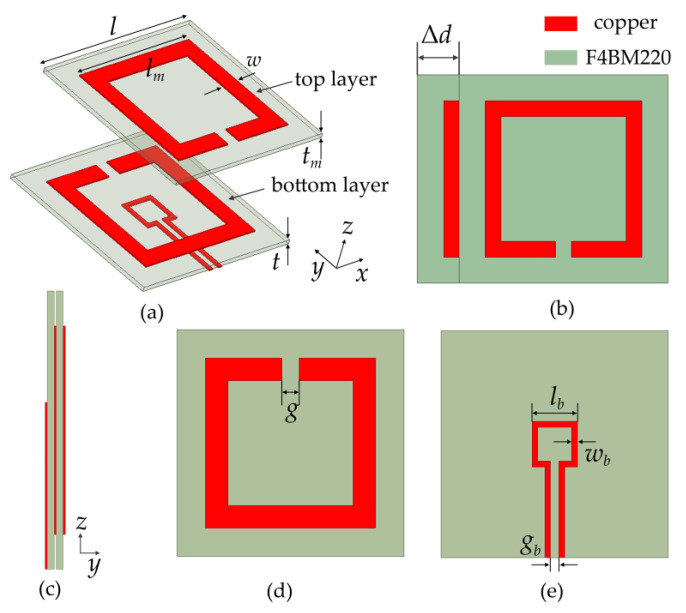
(**a**) Dismantling figure of the proposed sensor. (**b**) Perspective views of the proposed sensor with displacement Δ*d*. (**c**) Side view, (**d**) top view, and (**e**) bottom view of the proposed sensor. The design parameters are given in Table 1.

The sensor shown in Figure 1 consists of two layers of F4BM220 substrates (relative permittivity *ε*_r_ = 2.2, relative permeability *μ*_r_ = 1, and loss tangent *δ* = 0.003) printed with two metallic deep sub-wavelength-size SRRs in the opposite direction. All the parameters of the design are presented in Table 1. The relative displacement of two substrates can be produced by physically shifting two layers with respect to each other, and the lower surface of the bottom layer is printed with a metallic rectangular opening loop as the microwave energy feeding structure. The rectangular opening loop and SRRs are made of 0.035 mm thick copper, and the rectangular opening loop is directly connected with a 50-ohm Sub-Miniature Version A (SMA) radio frequency (RF) connector.

**Figure 2:**
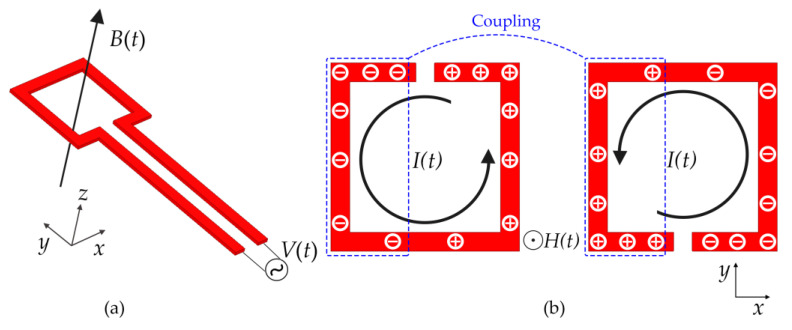
Schematic view of (**a**) the magnetic excitation generated by feeding structure and (**b**) the surface charge distribution and their corresponding surface current distribution for top-layer SRR and bottom-layer SRR, respectively.

**Figure 3:**
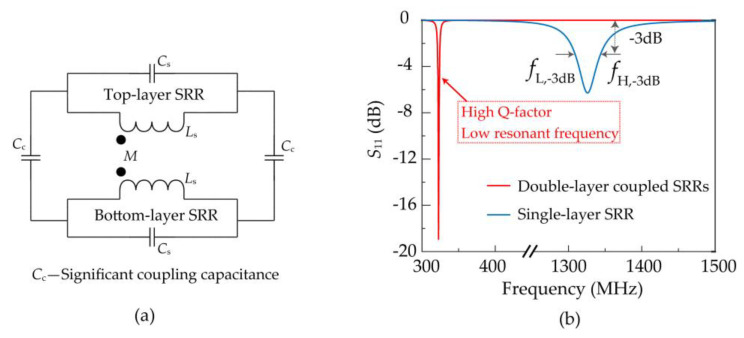
(**a**) The simplified *LC* equivalent circuit model of the double-layer coupled SRRs. (**b**) Simulated *S*_11_ of the double-layer coupled SRRs and single-layer SRR excited by the rectangular magnetic excitation loop.

The rectangular opening loop at the bottom of the lower substrate can be equivalent to a magnetic dipole, producing a magnetic field that oscillates perpendicular to the SRR plane [21,22]. Under the excitation of the magnetic field, two layers of SRR will induce the induced current in the same direction, which results in a positive mutual inductance *M*. Simultaneously, due to the opening of SRR restricting the flow of induced current, the positive and negative charges in the SRR are accumulated on the left and right sides, respectively. Figure 2 shows the process of establishing the surface charge distribution and their corresponding surface current distribution for SRR structures. Because the opening directions of the two layers of SRR are opposite, the positive and negative charges in the two layers overlap each other, which produces a significant mutual coupling capacitance *C*_c_. In the simplified *LC* equivalent circuit model as shown in Figure 3a, capacitance *C*_s_ and inductance *L*_s_ represent the distributed capacitance and self-inductance of a single SRR, respectively. The significant mutual coupling capacitance *C*_c_ and positive mutual inductance *M* provide a significant increase in the overall equivalent capacitance and inductance. Therefore, according to the resonant frequency Formula (1) of the *LC* resonant circuit [23], the significant equivalent capacitance and inductance of the double-layer coupled SRRs will lead to a smaller resonant frequency, thus greatly reducing the resonant electrical size of the metamaterial element.

(1) $$f=\frac{1}{2\pi \sqrt{LC}}$$

where *L* and *C* represent the equivalent inductance and equivalent capacitance in the metamaterial element’s equivalent circuit, respectively. The electromagnetic simulation software is employed to calculate the resonant characteristics of double-layer coupled SRRs and single SRR excited by the rectangular feeding loop as shown in Figure 3b, respectively. The resonant electrical size of the double-layer coupled SRRs is only 1/4 of that of the latter. At the same time, the *Q*-factor is higher than the latter owing to the double-layer coupled SRRs can store remarkably more electromagnetic energy compared with the single SRR [18]. The *Q*-factor can be calculated from the 3 dB bandwidth as:

(2) $$Q=\frac{f_{0}}{f_{H,-3\;\mathrm{dB}}-f_{L,-3\;\mathrm{dB}}}$$

where *f*_0_ is the resonant frequency of the sensor. Besides, *f*_H,−3 dB_ and *f*_L,−3 dB_ are the high and low frequencies at −3 dB on the *S*_11_ curve respectively. The *S*_11_ response is all lower than −3 dB in the band between *f*_H,−3 dB_ and *f*_L,−3 dB_, which can be considered that more than half of the microwave energy is fed into the sensor. The extremely small electrical size of the metamaterial element is the key factor for the miniaturization of the sensing structure in this design.

Due to the high *Q*-factor resonance of the coupled SRRs, the energy fed into the small feeding loop makes the coupled SRRs generate an enhanced resonant induced current. Hence, the input power is effectively coupled to the double-layer SRRs and radiated into the outer space, or consumed by the Ohmic resistance of the metals, without the need for additional matching networks. The use of the magnetic excitation method can avoid the guide wave structures with a large area of metal ground and thus obtains a miniaturized microwave single-port microwave device, which is the critical factor for the miniaturization of the feeding structure in this design.

The movable two layers of substrates can generate relative displacement along the *x*-direction, as shown in Figure 1b. The displacement will weaken the overlapping effect of the accumulated charge of different symbols of two layers of SRR, and also weaken the overlapping effect of the induced current in the same direction of two layers of SRR, which will reduce the coupling capacitance and mutual inductance simultaneously. The larger the relative displacement is, the more obvious the coupling capacitance and mutual inductance decrease. Thus, the resonant frequency shifting of the sensor becomes a function of displacements, which is the theoretical principle of the displacement sensor. According to the charge distribution analysis in Figure 2, the charge symbols at the corresponding positions of the upper and lower SRRs are opposite at resonance. Thus, the model of flat capacitors can be used for discussion. Suppose that the left or right half of an SRR carries a *Q* charge. According to Gauss theorem:

(3) $$\underset{\partial V}{∯}\vec{E}\cdot d\vec{S}=\frac{Q}{\varepsilon_{0}\varepsilon_{r}}$$

where *∂*V denotes the closed surface for integration. The voltage between two SRRs can be obtained at small displacement ∆*d:*

(4) $$U=E\sqrt{t_{m}^{2}+\Delta d^{2}}$$

Then, substitute Equations (3) and (4) into the definition of Capacitance (5):

(5) $$C=\frac{Q}{U}$$

The capacitance of the left or right double-layer SRR can be approximately expressed as a function of displacement:

(6) $$C=\frac{\varepsilon_{0}\varepsilon_{r}S}{\sqrt{t_{m}^{2}+\Delta d^{2}}}$$

where *S* represents the area of metal on one side of SRR. The displacement ∆*d* results in the decrease of the capacitance and the increase of the resonance frequency of the double-layer metamaterial element.

**Figure 4:**
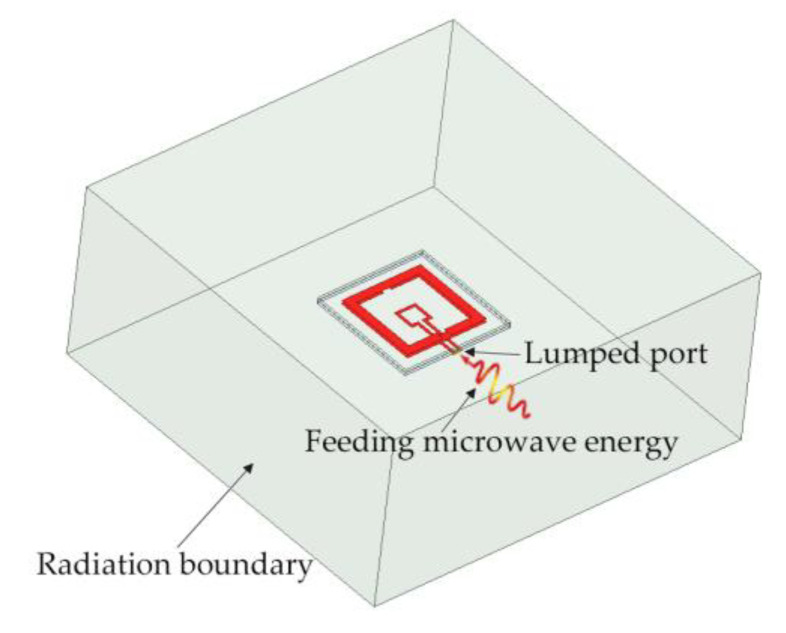
Schematic view of simulation settings.

To validate the proposed design of the electrically small-size displacement sensor, the sensor structure is modeled and simulated in the electromagnetic simulation software. Using the finite element method (FEM), the working principle of the sensor is analyzed and the displacement sensing process is simulated. In order to reduce the grid division and increase the computing speed, the 0.035 mm thick copper clad layer is replaced by perfect electric conductor (PEC) planes, and the coaxial feeding SMA RF connector is replaced by a 50-ohm lumped port. The two ends of the lumped port are connected with the long arms of the two ends of the rectangular feeding loop, respectively. The radiation boundary condition is set at a certain distance of not less than *λ*_0_/4 around the sensor structure to simulate the absorption of the energy radiated by the sensor as depicted in Figure 4.

**Figure 5:**
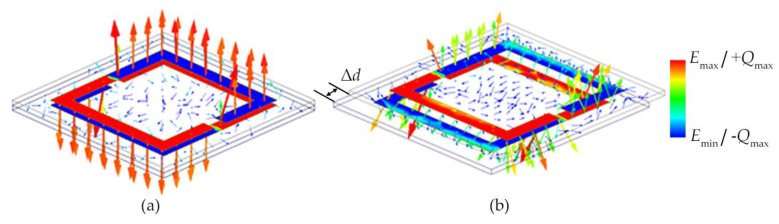
Simulated electric vector distributions between the double-layer coupled SRRs and charge distributions with (**a**) no displacement at 318.5 MHz and (**b**) 4 mm displacement at 444 MHz.

**Figure 6:**
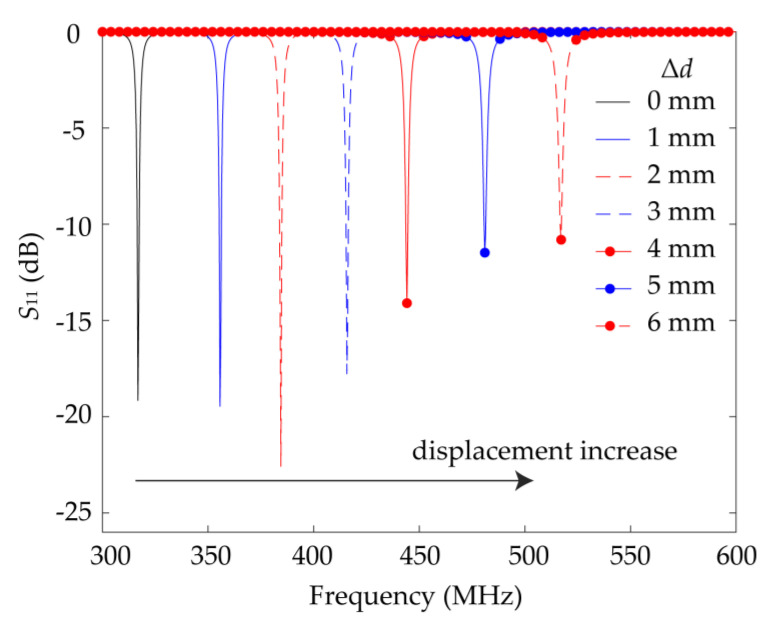
Simulated *S*_11_ curve of the designed sensor for different displacements Δ*d* = 0 mm to Δ*d* = 6 mm in steps of 1 mm.

The design theory is verified by the simulated field distribution. As shown in Figure 5, when no displacement between the double-layer SRRs is applied, there is a strong electric field that reaches 3.9 × 10^5^ V/m between the two SRRs from the positive charge aggregation to the negative charge aggregation, representing a strong coupling capacitance. After the introduction of a displacement, the electric field between the two SRRs still points from the positive charge aggregation to the negative charge aggregation, but the field strength is drastically weakened, which means that the coupling capacitance is significantly reduced. An increase in the displacement results in a decrease of the equivalent capacitance and inductance of the sensor and an increase in the resonant frequency. When the sensor resonates, the microwave energy fed through the rectangular feeding loop will be radiated or dissipated in the loss. Therefore, through the reflection coefficient of the lumped port in the simulation, the resonant absorption peak position of the sensor under different displacements can be distinguished. The reflection coefficients for different displacements are depicted in the simulation results of Figure 6. The resonant frequency of the sensor increases from 318.5 to 590 MHz as the displacement increases from 0 to 9 mm. Thus, an actual displacement value can be calculated from the measured resonance peak using the curve fitting method.

## 3. Experiment and Results

**Figure 7:**
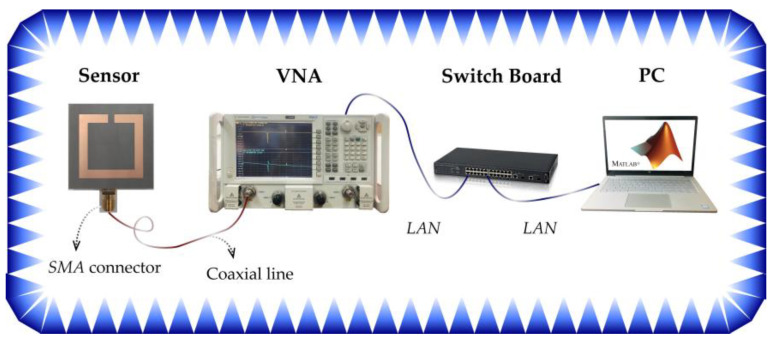
Whole view of the experimental system.

**Figure 8:**
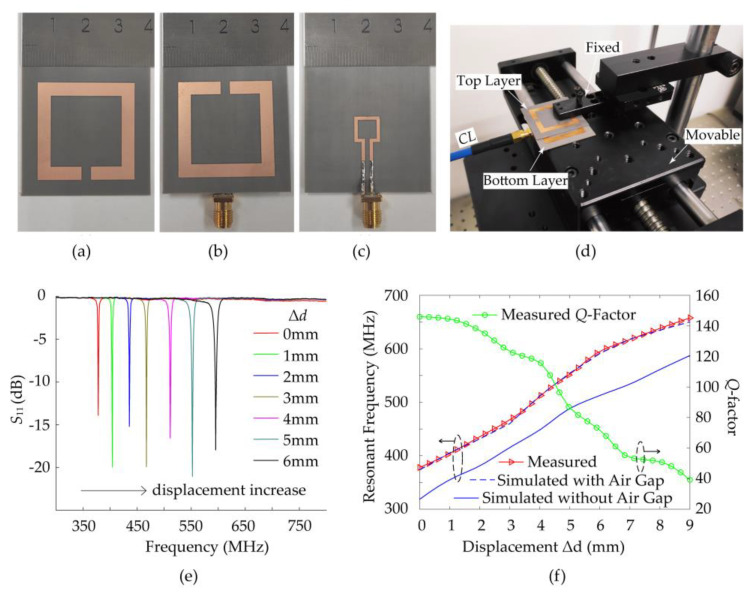
Picture and measured data of the fabricated sensor. (**a**) Top view of the upper substrate. (**b**) Top view and (**c**) bottom view of the lower substrate. (**d**) Measurement setup. (**e**) Measured *S*_11_ curve of the designed sensor for different displacements Δ*d* = 0 mm to Δ*d* = 6 mm in steps of 1 mm. (**f**) *Q*-factor and resonant frequency response of the fabricated sensor (air gap equals to 0.07 mm).

In order to verify the performance of the sensor for measuring displacement, the prototype has been fabricated as depicted in Figure 8a–c. Figure 7 shows the connection diagram in the experiment. Figure 8d shows a photograph of the measurement setup, which is composed of a computer numerical control (CNC) displacement table and a fixed strut. The two-part are connected to the two layers of the sensor respectively. The CNC displacement table and strut are on an optical table workstation to ensure the flatness of the displacement plane. Thereby the relative displacement of the two substrates of the sensor can be generated by the accurate one-dimension movement of the CNC displacement table. A 50-ohm SMA connector is mounted on the electrically small rectangular feeding loop to connect the fabricated sensor with an Agilent N5245A vector network analyzer (VNA) using a coaxial line (CL). The frequency spectrum of the *S*_11_ parameters is measured from 300 to 800 MHz. For post-processing data of VNA, the VNA and computer are connected to a local area network by a switchboard. Then, with the help of MATLAB software, the relationship between the resonant frequency of the sensor and the actual displacement value in the physical world can be obtained, so as to achieve the purpose of displacement sensing.

The single-port sensor’s resonant frequency can be detected by measuring its *S*_11_ parameters. In order to eliminate the error network between the test plane of the VNA and the test plane of the device under test (DUT), we use the “calibration” function of the VNA. After eliminating the error term, the electromagnetic characteristics of the sensor are displayed on VNA and the measured data are recorded on a dB scale. Figure 8d shows the measured and simulated *S*_11_ curve of the sensor for different displacements in steps of 1 mm. It can be noted that the measured resonant frequency for displacement at 0 mm (378.1 MHz) is found to be higher than the simulated data (318.5 MHz). In addition to the manufacturing accuracy error, one of the major reasons is found to be an air gap caused by the double-layer substrate not perfectly laminating [4]. The existence of the air gap will reduce the capacitance coupling between double-layer SRRs, which can explain that the measured value of resonant frequency is higher than the simulation value. Indeed, in order to make the movable layer of the sensor move steadily, an air gap is inevitably introduced between the two to make the movable layer of the sensor move steadily. Then, to determine the approximate thickness of the existing air gap, the thickness of the bottom layer and top layer with microcallipers are measured as *t*_1_ and *t*_2_, respectively. Afterward, the total thickness of the two layers after stacking together is measured as *t*_0_. The subtraction formula *t*_0_-*t*_1_-*t*_2_ is not equal to zero, but 0.06–0.08 mm. Thus, an air gap with an optimized thickness of 0.07 mm in the posterior simulation is utilized to match the measured result as shown in Figure 8f. According to the measurement results, the absolute sensitivity is calculated by:

(7) $$S=\frac{f_{\max}-f_{\min}}{\Delta d}$$

where *f*_max_ and *f*_min_ are the maximum and minimum resonant frequencies in the dynamic range, respectively, and ∆*d* is the difference between the maximum and minimum displacement in the dynamic range. The absolute sensitivity is up to 31.09 MHz∙mm^−1^. This absolute sensitivity *S* can be improved by increasing the difference between *f*_max_ and *f*_min_ under the same ∆*d*, i.e., reducing the size of the sensor to increase its resonant frequency. Besides, the relative sensitivity is calculated by:

(8) $$S_{0}=\frac{S}{f_{0}}$$

where *f*_0_ denotes the resonant frequency with no displacement. The relative sensitivity is up to 0.082 *f*_0_∙mm^−1^. Meanwhile, the thickness resolution *D* can be calculated as:

(9) $$D=\frac{D_{f}}{S}$$

where *D_f_* is the resolution of the VNA. When the resolution of the VNA is 1 MHz, the minimum resolution of the sensor for the displacement is 32.16 μM, which means that the sensor can recognize a difference of 32.16 μM displacement, whose electrical dimension is only 4.05 × 10^−5^ *λ*_0_ at 378.1 MHz. This displacement resolution *D* can be improved by increasing the resolution of VNA. As shown in the measured data in Figure 8e–f, the sensor has a wide dynamic measurement range, but the resonance peak resolution will be reduced as the *Q*-factor decreases when measuring large displacements, which is due to the decrease of electromagnetic field energy binding capacity of the double-layer coupled SRRs after shifting with respect to each other. After measuring, the *Q*-factor of the sensor decreases from 143.7 to 40 as the displacement increases from 0 to 9 mm. Table 2 provides a comparison between the proposed displacement sensor and several previously published microwave displacement sensors. According to Table 2, our proposed sensor has smaller and simpler structures with high sensitivity.

## 4. Discussion

In the above, the design of an electrically small-size sensor is proposed to implement the displacement sensor into a deep sub-wavelength structure. Based on this platform, we discussed the influence of substrate thickness, the robustness of excitation structure, and the expansion of displacement sensing dimension. The purpose of these discussions is to find a broader prospective application of the displacement sensing methodology.

### 4.1. Influence of Substrate Thickness

**Figure 9:**
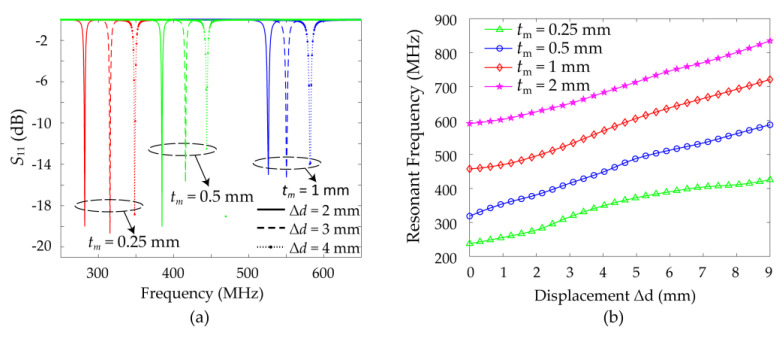
(**a**) Simulated *S*_11_ of the sensor for different displacements Δ*d* = 2 mm to Δ*d* = 4 mm in steps of 1 mm under different substrate thicknesses. (**b**) Simulated resonant frequency of the sensor under different thicknesses of the upper substrate.

According to the above analysis, in addition to the displacement, the thickness of the sensing structure will also have a significant impact on the resonance of the sensor. From Formula (6) of the coupling capacitance analysis, it can be seen that the greater the thickness *t* of the substrate, the smaller the influence of displacement on coupling capacitance, and the corresponding sensitivity will be lower. The smaller the thickness, the greater the influence of displacement on the coupling capacitance, and the corresponding sensitivity will be higher. However, the increase of the sensitivity caused by the thickness of the substrate results in the decrease of the linearity of the displacement resonance frequency curve. In order to verify the analysis, displacement signal responses under different thicknesses of the upper substrate condition are compared. Figure 9 demonstrates resonant frequencies of the sensor under different thicknesses of the top-layer substrate calculated by FEM. A thick substrate will increase the working frequency, and decrease the sensitivity of the sensor, but at the same time, the linearity will increase. Therefore, it is possible to use the same sensor to satisfy a variety of displacement testing requirements by choosing different thicknesses of the upper substrate. In the test environment pursuing the linearity of measurement signals, a thicker substrate is a better choice, however, in the test environment pursuing the sensitivity of measurement signal, a thinner substrate is a better choice.

**Figure 10:**
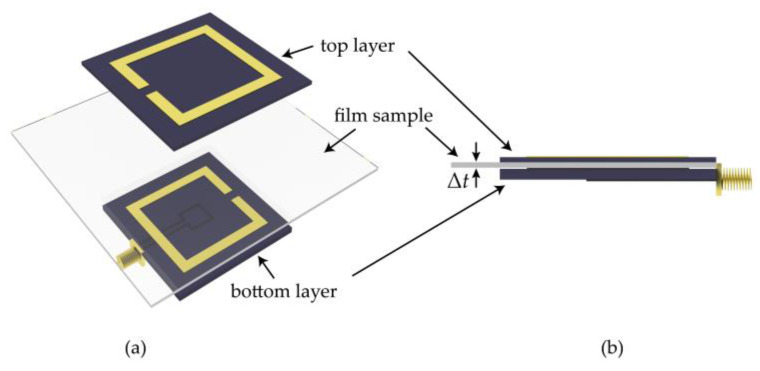
(**a**) Dismantling figure of the proposed thickness sensor, whose dimensions are the same as those of the above displacement sensor. (**b**) Side view of the proposed thickness sensor. The two layers of the sensor form a sandwich structure with the film sample.

**Figure 11:**
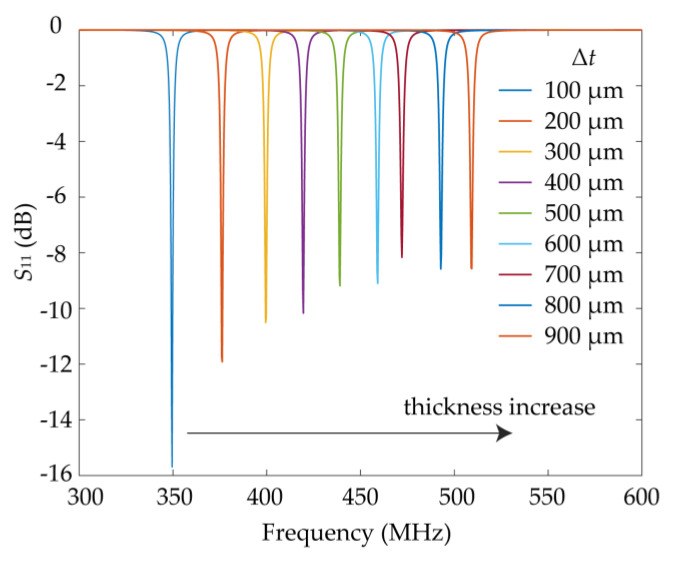
Simulated *S*_11_ curve of the designed sensor for different thicknesses of the PVA film. The thickness range in the simulation setting is Δ*t* = 100 μm to Δ*t* = 900 μm in steps of 100 μm.

In addition, monitoring the thickness of thin-film samples is another essential demand of industrial applications. Based on the analyses on the capacity coupling of the double-layer SRR, here, we also provide a compact thickness sensor of high sensitivity based on the electrically small-size sensor presented in Figure 10. To demonstrate the functional expansibility of the sensing method, the size of the thickness sensor is selected to be the same as that of the above displacement sensor. As an important non-toxic and biocompatible polymer, polyvinyl alcohol (PVA) is used to make emulsifiers, paper coating, adhesive, glue, etc., whose relative permittivity at microwave frequency is 2.2. Thus, a thin polyvinyl alcohol film is chosen as the sensing sample in the simulations. The thin film is sandwiched between the two-layer SRRs. By proper clamping, the sliding of the film and the air gap between film and sensing structure can be avoided. The results shown in Figure 11 show that the resonant dip blueshifts as the film thickness increases. The resonant frequency of the thickness sensor increases from 349 to 509 MHz as the thickness of the film clamped between the two SRRs increases from 100 to 900 μM. The sensitivity at the dynamic range between 100 to 900 μM is calculated by:

(10) $$S=\frac{f_{\max}-f_{\min}}{\Delta t}$$

where *f*_max_ and *f*_min_ are the maximum and minimum resonant frequencies in the dynamic range, respectively, and ∆*t* is the difference between the maximum and minimum thickness of the film in the dynamic range. The sensitivity is up to 0.2 MHz∙μM^−1^. When the resolution of the VNA is 1 MHz, the minimum resolution of the thickness sensor for the thin film thickness is 5 μM, which means that the sensor can recognize a difference of 5 μM thickness, whose electrical dimension is only 5.8 × 10^−6^ *λ*_0_ at 350 MHz.

### 4.2. Robustness of Excitation Structure

**Figure 12:**
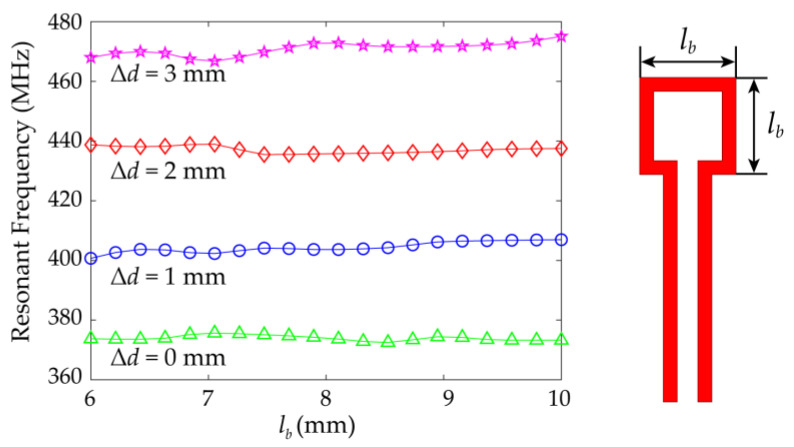
Simulated resonant frequency of the sensor with air gap under different sizes of the magnetic excitation rectangular opening loop.

**Figure 13:**
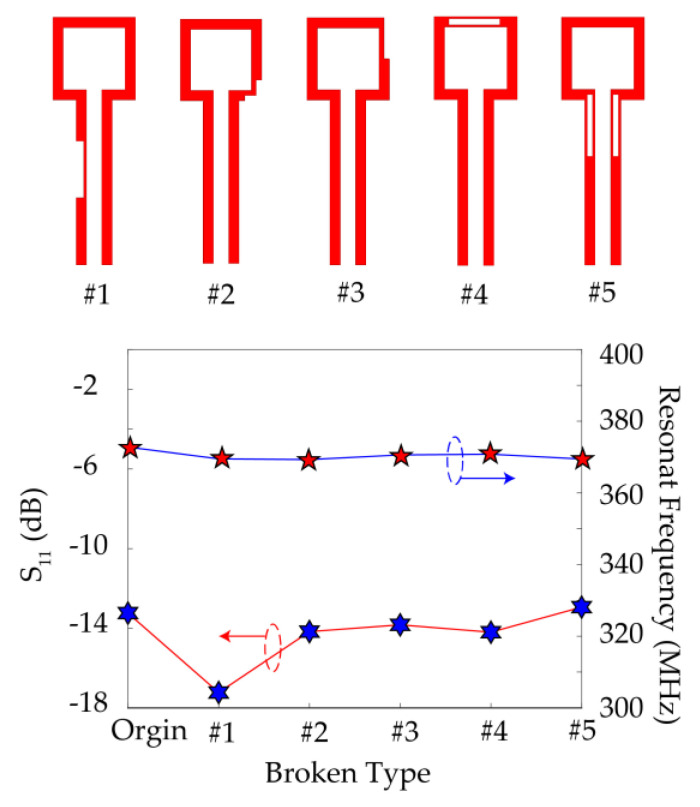
Simulated reflection coefficients and resonant frequency of the sensor with random damage on the feed excitation structure.

Due to the exchange of energy between the rectangular opening loop and the high-*Q* metamaterial element by magnetic coupling, this feeding excitation method has strong robustness. In other microwave sensors in literature, microstrip line and coplanar waveguide are often used as the feeding structure. This kind of feeding structure needs to be optimized to meet the requirements of microwave network matching so that the microwave energy can be coupled to the sensing structure. The strip size of microstrip lines or the size of coplanar waveguides will greatly affect the performance of the sensor. However, for the above-proposed microwave sensor, the size of the rectangular opening loop is independent of the performance of the sensor in a certain range as shown in the simulation results in Figure 12. In addition, we verify the resonant characteristics of the sensor in the presence of random damage in the feed excitation structure. This random damage simulates the abrasions of the sensor in the process of long-term use. A group of notches is set on the rectangular loop, and FEM is used to calculate the *S*_11_ curve of the sensor. As shown in Figure 13, the resonant frequency and depth remain stable when the rectangular loop is damaged but not cut off. This further proves the strong robustness of the feed excitation structure in microwave sensor applications.

### 4.3. Expansion of Displacement Sensing Dimension

**Figure 14:**
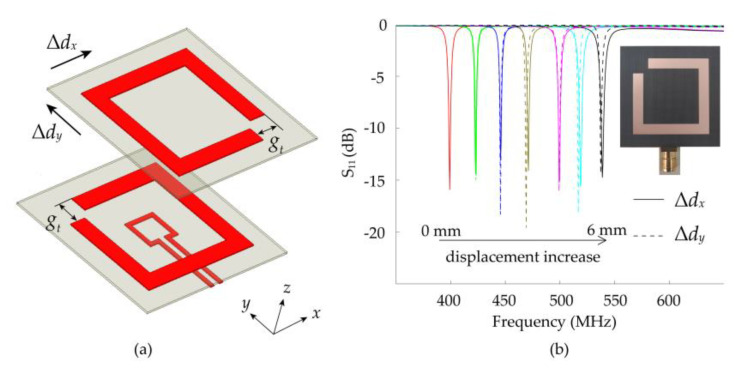
(**a**) Dismantling figure of the proposed bidirectional displacement sensor. (**b**) Measured *S*_11_ curve of the designed sensor in two direction with different displacements Δ*d* = 0 mm to Δ*d* = 6 mm in steps of 1 mm and the picture of the fabricated sensor.

**Figure 15:**
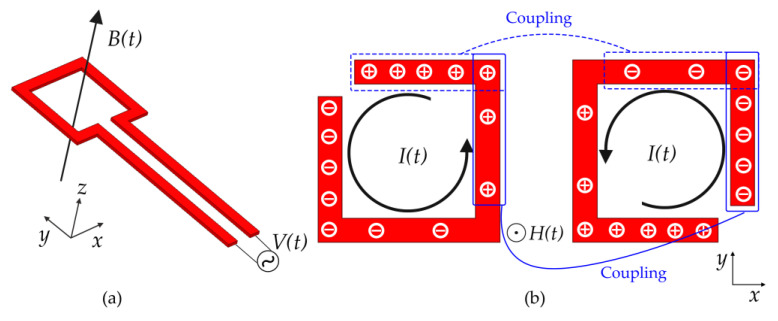
Schematic view of (**a**) the magnetic excitation generated by feeding structure and (**b**) the surface charge distribution and their corresponding surface current distribution for top-layer SRR and bottom-layer SRR of the bidirectional displacement sensor, respectively.

Furthermore, when the gap is opened in the corner of the SRRs and the gap of the two-layer SRRs is centrosymmetric distribution as shown in Figure 14a, the proposed 1-D displacement sensor can be easily expanded for sensing displacement in two orthogonal directions without increasing its physical size. In contrast to the proposed 1-D displacement sensor, the distribution of coupling capacitance in the *x*- and *y*-axis directions is the same due to the two centrosymmetric gaps, as shown in Figure 15. Within a certain range of displacement, the weakening of the coupling effect caused by the *x*-direction displacement is the same as that caused by the *y*-direction displacement. Therefore, the sensor has the same sensitivity in the two dimensions. This feature enables the proposed sensor to sense the *x*-axis (rep. *y*-axis) displacement when the *y*-axis (rep. *x*-axis) displacement is fixed. In this way, the application scenarios of the 1-D displacement sensor are broadened. In order to verify the performance of the sensor for measuring bidirectional displacement, the prototype has been fabricated as depicted in Figure 14b and tested in the experimental system mentioned above. The measured reflection coefficients prove the theoretical analysis. As the displacement increases, the blue shift of the resonant frequency in both directions is almost the same, and the sensitivity is 23.14 MHz∙mm^−1^ (0.058 *f*_0_·mm^−1^) in the dynamic range of 0–6 mm.

## 5. Conclusions

In this paper, a microwave displacement sensor with an electrically small size based on the shift of resonant frequency has been designed, fabricated, and tested. The relative displacement between two surfaces can be measured using the proposed sensor. The size of the sensor can be significantly reduced by utilizing the excitation structure of the rectangular feeding loop and the sensing structure of the metamaterial element, i.e., the strongly coupled double-layer SRRs. Additionally, based on the proposed sensing method, the monitoring accuracy of the displacement can be further improved by increasing the resonant frequency of the sensor. The sufficiently compact design of the proposed sensor is conducive to the miniaturization and integration of sensor equipment. Simultaneously, the strong robustness and functional expansibility of the sensor are also demonstrated, which expands the application prospects of the sensing method in industrial fields.

## Figures and Tables

**Table 1:** Design parameters and their values.

Parameters	*l*	*l_m_*	*t*	*g*	*l_b_*	*w_b_*	*g_b_*	*t_m_*
**Value (mm)**	40	30	1	3	8	1	1	0.5

**Table 2:** Comparisons of the 1-D displacement microwave sensor.

Ref.	Type	Operating Frequency	Sensitivity	Range (mm)	*Q*	Size (mm)	Electrical Size (*λ*_0_^2^)	Measurement Dimension
[7]	CPW with SRR	1.17 GHz	23.6 dB·mm^−1^	1.2	19	30.3 × 15.3	7.06 × 10^−3^	1-D
[8]	Microstrip line with SRRs	1.6 GHz	0.029 *f*_0_·mm^−1^	5	53	26.3 × 30.9	4.41 × 10^−2^	1-D
[9]	CPW with DGS	3.8 GHz	0.228 *f*_0_·mm^−1^	3	34	42.8 × 26.1	1.79 × 10^−1^	1-D
[14]	Corrugated SIW	2.4 GHz	0.011 *f*_0_·mm^−1^	15	65	80 × 50	2.56 × 10^−1^	1-D
[15]	Microstrip line with SRRs	5.2 GHz	0.096 *f*_0_·mm^−1^	5	226	7.5 × 7.5	1.69 × 10^−2^	1-D
This work	Magnetic dipole with SRRs	378 MHz	0.082 *f*_0_·mm^−1^	9	143.7	40 × 40	2.5 × 10^−3^	1-D

## Data Availability

The data presented in this study are available on request from the corresponding author.
